# Advances in primary large B-cell lymphoma of immune-privileged sites

**DOI:** 10.3389/fimmu.2025.1533444

**Published:** 2025-02-26

**Authors:** Liao Wang, Meiru Guo, Shuling Hou

**Affiliations:** ^1^ Shanxi Bethune Hospital Cancer Center Lymphoma Department, Shanxi Academy of Medical Sciences, Tongji Shanxi Hospital, Third Hospital of Shanxi Medical University, Taiyuan, China; ^2^ Tongji Hospital, Tongji Medical College, Huazhong University of Science and Technology, Wuhan, China; ^3^ Third Hospital of Shanxi Medical University, Shanxi Bethune Hospital, Shanxi Academy of Medical Sciences, Tongji Shanxi Hospital, Taiyuan, China

**Keywords:** primary large B-cell lymphoma of immune-privileged sites, pathology, diagnosis, treatment, prognosis

## Abstract

Primary large B-cell lymphoma of immune-privileged sites (IP-LBCL) encompasses a spectrum of relatively rare aggressive B-cell lymphomas, such as primary central nervous system lymphoma (PCNSL), primary testicular large B-cell lymphoma (PTL), and primary vitreoretinal large B-cell lymphoma (PVRL). Macroscopically, the development of IPI-LBCL may be associated with the dysfunction of meningeal lymphatic vessels (mLVs) and the perivascular channel system formed by astrocytes. Microscopically, mutation in MYD88 and CD79B genes plays a pivotal role in the pathogenesis of IP-LBCL. Pathological examination remains the cornerstone for establishing a diagnosis of IP-LBCL. Moreover, traditional imaging is now supplemented by a suite of advanced diagnostic methods, including cytological, genetic, immunological, multiple omics, and molecular biological, which collectively enhance the diagnostic accuracy of IP-LBCL. Despite these advancements, the high recurrence rates and attendant high mortality rates pose significant challenges to achieving long-term survival in IP-LBCL patients. However, the emergence of novel therapeutic agents, such as Bruton’s tyrosine kinase inhibitors (BTKi), immune checkpoint inhibitors, immunomodulators, and anti-CD19 chimeric antigen receptor T (CAR-T) cell therapy, has offered promising new avenues for the treatment of IP-LBCL, demonstrating remarkable anti-tumor efficacy in recent years. This review delves into the epidemiology, pathogenesis mechanisms, diagnosis approaches, therapeutic strategies, and prognosis factors associated with IP-LBCL. It meticulously examines the parallels and divergences between the National Comprehensive Cancer Network (NCCN) and European Society for Medical Oncology (ESMO) guidelines, enhancing the professional comprehension of the complexities inherent to IP-LBCL.

## 1 Introduction

Primary large B-cell lymphoma of immune-privileged sites (IP-LBCL) was introduced in the 5th edition of the World Health Organization Classification of Lymphoid Neoplasms in 2022. This classification delineates IP-LBCL as a distinct group of aggressive B-cell lymphomas that primarily manifest in the central nervous system, vitreous retina, or testes. This group encompasses primary central nervous system lymphoma (PCNSL), primary testicular large B-cell lymphoma (PTL), and primary vitreoretinal large B-cell lymphoma (PVRL). Given the significant biological similarities and shared intravascular microenvironment between intravascular large B-cell lymphoma (IVLBCL) and IP-LBCL, which may also be considered a site of immune privilege, IVLBCL has been incorporated into the IP-LBCL category (1). Furthermore, primary cutaneous diffuse large B-cell lymphoma (DLBCL) leg-type and primary breast or adrenal DLBCL are also posited to be variants of IP-LBCL. The pathogenesis of IP-LBCL is intricate due to physiological barriers such as the blood-brain barrier (BBB), blood-retina barrier, and blood-testis barrier, leading to poor treatment responses, high recurrence rates, and unfavorable prognoses. Accurate diagnosis and effective treatment protocols are essential for managing IP-LBCL patients. This review aims to synthesize the current research on IP-LBCL, offering a robust foundation for clinical practice to provide a reliable reference for clinical work.

## 2 Epidemiology

IP-LBCL represents a rare and highly aggressive subset of extranodal non-Hodgkin lymphomas (NHLs) from non-germinal centers. The annual incidence rate of PCNSL is 0.4/100,000 (2), constituting 4% to 6% of all extranodal lymphomas. While PCNSL can manifest at any age, its incidence escalates with increasing age, peaking at a median age of approximately 67. Among those over 70, the condition affects approximately 4 cases per 100,000 individuals (3). PTL comprises 1-2% of all NHLs and accounts for 4% of extranodal NHLs (4). It is more prevalent in the elderly male population, with a median age at diagnosis ranging from 66 to 68 years (5). PVRL usually occurs in immunocompetent adults in their 50s, with a slight female predominance but no racial predilection. PVRL incidence is estimated to be 50 cases annually in the United States. PVRL represents ~5% of the patients registered in the French database for oculocerebral lymphomas and accounts for 10 new cases annually (6). There is a lack of incidence statistics for PVRL in other regions. Apart from immunosuppression related to the human immunodeficiency virus (HIV), there is a lack of information regarding the incidence, geographic or racial disparities, and potential risk factors for the broader category of IP-LBCL.

## 3 Pathology

From a macro perspective, immune-privileged sites typically lack lymphatic tissue. Whether lymphomas originate in these organs or develop outside and subsequently home to them is a contentious and ongoing area of research. Currently, there is a paucity of research on the pathogenesis of PTL. Regarding the mechanisms of PCNSL and PVRL, some studies suggest that PCNSL and PVRL may originate outside the CNS and then migrate to the CNS or the eye, proliferating in the immunologically permissive microenvironment (6).

Identifying meningeal lymphatic vessels (mLVs) in the dura mater challenges this traditional view. These anatomical structures serve as potential drainage pathways for molecular clearance and as conduits for immune cells to access the peripheral lymphatic system from the cerebrospinal fluid. Dysfunction of mLVs has been correlated with impaired brain waste clearance, potentially contributing to the development of PCNSL (7). Moreover, the glial lymphatic system, implicated in waste removal, fluid/ion homeostasis, inflammatory response, and immune surveillance in the CNS, appears to play a significant role in the proliferation and spread of lymphomas (8). This system includes perivascular channels formed by astrocytes, such as the Virchow-Robin perivascular hiatus, facilitating the flow of cerebrospinal fluid from the subarachnoid space and interstitial fluid into the dural sinuses. Additionally, a growing body of research indicates that in IP-LBCL, inhibitory microenvironmental factors, including T cell depletion and macrophage immune function downregulation, substantially influence tumor cell proliferation, immune escape, and drug resistance.

**Figure 1:**
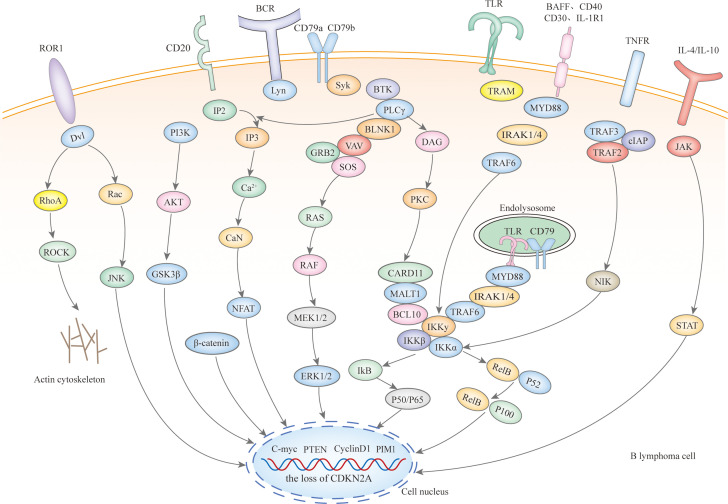
The signaling mechanisms within IP-LBCL.

Microscopically, IP-LBCL is characterized by genomic instability affecting multiple signaling pathways and cellular processes (**Figure 1**), including nuclear factor-κB (NF-κB), B-cell receptor (BCR), Toll-like receptor (TLR), JAK-STAT signaling pathway, mitogen-activated protein kinase signaling pathway, DNA damage response, apoptosis, cell-cycle regulation as well as tumor immune microenvironment (TME) dysfunction, notably including the signature mutations MYD88 L265P (67%), CD79B (63%), and CDKN2A deletions (83%) (9).

Mutations in the myeloid differentiation primary response gene 88 (MYD88) and the B-cell receptor beta chain (CD79B) are pivotal in the pathogenesis of IP-LBCL. Staudt’s team has proposed the ‘gene heptad’ that classifies DLBCL with co-mutations in MYD88 L265P and CD79B as the MCD type, while Shipp’s team ‘gene quintet’ suggests that C5 corresponds to the MCD type, hence IP-LBCL is also referred to as MCD/C5 subtype lymphomas. These mutations in MYD88 and CD79B enhance B-cell survival and proliferation by activating TLR and BCR signaling, leading to constitutive activation of the NF-κB pathway downstream (10). It should be noted that Roschewski et al. identified the “My-T-BCR” supercomplex in PCNSL. This supercomplex, named for its co-localization of MYD88 with TLR9 and BCR, serves as a site for NF-κB activation. Receptor tyrosine kinase-like orphan receptor 1 (ROR1), typically absent or minimally expressed in normal tissues, and over-expressed in R/R DLBCL, interacts with Wnt-related proteins, promoting tumor cell proliferation, activation, and epithelial-to-mesenchymal transformation through the Wnt/β classical pathway and PI3Kδ/AKT/mTOR non-classical Wnt pathway (11). The role of ROR1 in the development of IP-LBCL warrants further investigation. Deletions of the cell cycle-dependent kinase inhibitor 2A (CDKN2A) gene and the human leukocyte antigen (HLA) locus also contribute to developing IP-LBCL. CDKN2A, a critical tumor suppressor gene located in the 9p21 region of the human chromosome, when deleted, leads to loss of cell cycle regulation and an increased risk of cancer development. Although TP53 mutations are rare in IP-LBCL, they can disrupt the p53 pathway through upstream CDKN2A loss, which is often near-uniform and involves a double allele.

**Figure 2:**
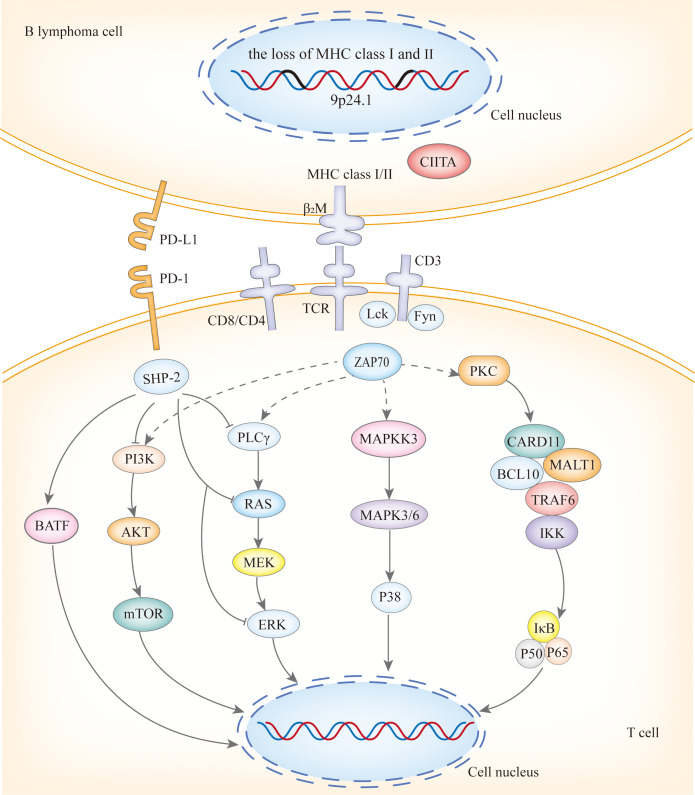
The immune escape mechanisms of IP-LBCL.

Copy number alterations(CNAs) at 9p24.1/PD-L1, along with translocations with concomitant protein overexpression, the loss of HLA class I and II expression, and the loss of HLA loci, form the basis of immune escape in PCNSL and PTL (**Figure 2**), with the latter playing a significant role (12). PCNSL notably demonstrates a higher frequency of focal deletions in the HLA-D (6p21) locus, suggesting a potential mechanism of immune evasion (9). Immune escape and sustained signaling are hallmark features of PTL, encompassing structural rearrangements of the core components of antigen presentation, including CIITA, B2M, and HLA loci, as well as programmed death ligands 1 (CD274) and 2 (PDCD1LG2). Somatic mutation enrichment within NF-κB pathway genes - namely MYD88, CD79B, NFKBIZ, BCL10, and MALT1- is also a prominent feature of PTL (4). These genetic alterations within the NF-κB pathway underscore the significance of immune evasion and sustained signaling in the pathogenesis of PTL (13). PVRL’s mutational spectrum, which includes activation of the toll-like receptor and B-cell receptor pathway alongside the loss of CDKN2A, substantiates its close affiliation with other IP-LBCL (14). Similarly, analogous genetic alterations in IVLBCL, primary cutaneous DLBCL leg-type, and primary breast or adrenal DLBCL support their inclusion within the IP-LBCL classification.

## 4 Diagnosis

### 4.1 Pathological examination

Pathological examination is considered the gold standard for diagnosing IP-LBCL. The combination of cytological, immunological, cytogenetic, and molecular biological assays enables a precise diagnosis of IP-LBCL. The pathological, molecular, and genetic changes of IP-LBCL are detailed in the **Supplementary Table 1**.

Stereotactic biopsy is the preferred approach for PCNSL. Approximately 95% of PCNSL have exhibited a DLBCL pathological subtype, predominantly of non-germinal center origin. The remaining cases include marginal zone lymphoma (MZL), anaplastic large cell lymphoma (ALCL), Burkitt lymphoma (BL), lymphoblastic lymphoma (LBL), mature T/NK-cell lymphoma (T/NK), and Hodgkin lymphoma (HL).

In the diagnosis of primary testicular lymphoma (PTL), orchiectomy is favored over fine needle aspiration and testicular biopsy due to the availability of intact tissue samples. Approximately 80% to 90% of PTL cases are classified as DLBCL; other pathologic types are predominantly Burkitt and Burkitt-like lymphoma (BL). Mantle cell, NK/T-cell, and follicular testicular lymphoma (FL) are observed less frequently.

PVRL is occasionally classified as a subtype of PCNSL. A vitreous biopsy is the preferred method for diagnosing PVRL. Lymph node or local lesion biopsy is the preferred method for diagnosing IVLBCL, primary cutaneous DLBCL leg-type, and primary breast or adrenal DLBCL.

### 4.2 Laboratory examination

Research has demonstrated that a κ to λ free immunoglobulin light chains ratio exceeding 3.0 in Cerebrospinal Fluid (CSF) patients with PCNSL can be a provisional threshold for identifying clonal light chain restriction. Additionally, the CSF levels of cytokines IL-10, IL-6, IL-10/IL-6 ratio, and C-X-C chemokine ligand 13 (CXCL 13) have been identified as potential diagnostic markers for PCNSL. Studies have indicated that the diagnostic threshold value of IL-10 was 0.43 pg/mL, yielding a sensitivity of 96.3% and a specificity of 66.67%. While IL-6 alone was not significant for diagnosing PCNSL, the IL-10/IL-6 was significant, with a critical value of 0.21, a sensitivity of 81.48%, and a specificity of 80.95% (15). Furthermore, CXCL 13, which plays an essential role in B-cell homing, has been recognized as an independent diagnostic marker and can be utilized alone or in conjunction with IL-10 (16). A ROC curve analysis assessed the diagnostic potential of CXCL 13 as a biomarker for CNS lymphoma, revealing that CXCL 13 concentrations above 90 pg/mL had a sensitivity of 69.9% and a specificity of 92.7% for detecting CNS lymphoma.

IL-6 and IL-10 levels in the aqueous humor or vitreous body have been implicated in the diagnosis of PVRL (17). A systematic review has demonstrated that an IL-10/IL-6 ratio of 1 or higher is highly sensitive for identifying patients with PVRL (18). Notably, the diagnostic utility of the IL10 to IL6 ratio differs between PCNSL and PVRL, suggesting that further investigation is warranted to elucidate the underlying reasons for this discrepancy.

### 4.3 Imaging

For diagnosing PCNSL, contrast-enhanced magnetic resonance (MRI) and diffusion-weighted (DWI) are the preferred imaging modalities. Most PCNSL cases manifest as isolated supratentorial lesions affecting the white matter. However, multifocal disease, involvement of the infratentorial compartments, and spinal cord involvement are also not uncommon occurrences (19). Research has shown that the notch sign, T2 necrosis sign, reef sign, and peritumoral white matter softening sign are closely associated with PCNSL (20). Concurrently, advanced MRI techniques, such as dynamic contrast-enhanced MRI and dynamic magnetic susceptibility contrast-perfusion MRI, offer distinctive insights into tumor biology.

Optical coherence tomography (OCT) is a valuable tool for accurately identifying the unique features and location of PVRL, which is essential for its diagnosis. OCT examinations reveal hyperreflective spots within the posterior vitreous cavity, with punctate or clustered hyperreflective foci that may infiltrate all layers. In severe cases, the retinal layers may become indistinguishable. Additionally, vertically oriented hyperreflective lesions within the retinal neuroepithelium are frequently observed. These lesions may also encircle the retinal pigment epithelium (RPE), creating a double layer between the RPE and Bruch’s membrane.

Ultrasonography is the preferred diagnostic modality for PTL and can also be utilized for the exclusionary diagnosis of PVRL. PVRL is characterized by infiltration along the spermatic cord and gonadal veins to the retroperitoneum and diffuse thoracic and abdominal infiltrates (21).

Fundus autofluorescence (FA) and indocyanine green angiography (ICGA) are diagnostic tools PVRL patients use. Distinct clusters of round hypofluorescent foci are observed in both the early and late stages of FA, as reported by Venkatesh et al. Proposed capillary detachment is another feature of FA in PVRL (22). The primary characteristic of ICGA is hypoblue light (23), and small, round hypofluorescent foci may disappear in advanced stages. FA is a non-invasive test for PVRL, where tumor cells infiltrating the neuroretina exhibit high autofluorescence, and lesion regression can be tracked by hypofluorescence (22).

Enhanced computed tomography (CT) of the chest, abdomen, and pelvis is crucial for determining the extent of IP-LBCL and distinguishing between primary and secondary lesions. Compared to CT, 18F-fluorodeoxyglucose positron emission tomography (18F-FDG PET) has a higher diagnostic value. A meta-analysis of 967 patients from 29 studies revealed that the weighted mean combined sensitivity, specificity, positive predictive value, negative predictive value, and diagnostic ratio for 18F-FDG PET were 87% (95% CI, 83%–90%), 85% (95% CI, 81%–88%), 84% (95% CI, 81%–88%), 87% (95% CI, 84%–90%), and 29.78 (95% CI, 18.34–48.35), respectively (24). However, the diagnostic value of both tests is not without limitations. A large international multicenter cohort study indicated a false-positive rate of up to 5.8% for whole-body PET-CT in the preliminary diagnosis of extracranial invasion in CNS DLBCL patients (25). Kim et al. also found that 13.5% of PCNSL patients showed low FDG uptake on PET, which is associated with negative expression of MUM1, a critical regulatory protein in B cell development and tumorigenesis (26). Therefore, caution must be exercised regarding false positives and negatives when diagnosing PCNSL with PET-CT. Pathological examination remains the gold standard for diagnosis.

PET serves a dual role in assessing treatment efficacy and predicting patient outcomes in CNS lymphoma. A prospective multicenter study evaluated the utility of 18F-FDG PET in treating CNS lymphoma, revealing that baseline cerebellar metabolism and the sum of metabolized tumor volume (sumMTV) for up to five lesions were predictive of chemotherapy response in PCNSL patients. Higher average standardized uptake values (SUV) and lower sumMTV in the cerebellum were significantly associated with favorable response groups in the study (average SUV: 6.4 [5.7–7.5] vs. 5.4 [4.6–6.5], p = 0.04, and sumMTV: 5 [1.8–10.8] vs. 17.1 [5.3–19.9], p = 0.01, respectively) (27), This suggests that elevated average SUV and reduced sumMTV in the cerebellum are indicative of improved treatment outcomes. Additionally, another prospective study reported that a high maximum standardized uptake value (SUVmax) correlated with decreased progression-free survival (PFS), with a median PFS of 3.4 months for patients with SUVmax >20 and 10.8 months for those with SUVmax <20 (28).

### 4.4 Fundus photography

An accurate ophthalmologic evaluation can discern infiltrative patterns of cells within the vitreous, presenting as sheets or clumps, and multifocal creamy-white lesions in the outer retina, characteristic findings in PVRL (23).

### 4.5 Biomarkers

Circulating tumor DNAs (ctDNAs), which are DNA fragments deriving from apoptotic, necrotic, or secreted tumor cells, serve as biomarkers in oncological research (29) (14). Compared to traditional tissue biopsies, ctDNAs offer the advantages of being non-invasive, requiring only a tiny sample, real-time, and allowing for multiple tests, making them particularly suitable for patients from whom samples cannot be obtained. On the other hand, ctDNAs carry the genomic information of tumor cells, reflecting the status of IP-LBCL. Detection of ctDNAs facilitates the acquisition of IP-LBCL’s genetic profiling, aiding in the selection of appropriate targeted therapies, but also enables real-time monitoring of tumor burden through changes in ctDNA levels, predicting tumor recurrence and surveillance of minimal residual disease. In brain tumor patients, the concentration of ctDNA in CSF exceeds that found in plasma, endowing it with heightened sensitivity for detecting CNS lesions (30). A CSF NGS analysis of 11 newly diagnosed PCNSL patients undergoing ibrutinib-based therapy demonstrated that ctDNAs could effectively monitor tumor burden and evaluate treatment response (29). Furthermore, the clinical utility of ctDNA in aqueous humor and vitreous fluid has also been established to diagnose and monitor PVRL (31). Similarly, changes in the microRNAs (miRNAs) levels also reflect crucial biological information about IP-LBCL, providing valuable reference information for evaluating treatment efficacy, assessing prognosis, monitoring drug resistance, and personalized treatment, thereby supporting clinical management and evidence-based decision-making. Several studies have employed real-time quantitative polymerase chain reaction (qRT-PCR) quantified miRNAs in the CSF of PCNSL patients, identifying significantly elevated expression levels of miR-19b, miR-21, and miR-92a relative to controls (32). Moreover, studies have indicated that miR-326 was a key driver of B-cell proliferation, and miR-6513-3p had the potential to serve as an auxiliary tool for the diagnosis of PVRL (33).

Hernandez-Verdin’s team conducted a comprehensive multi-omics analysis of PCNSL, encompassing whole-exome sequencing, RNA sequencing (RNA-seq), methylation sequencing, and clinical profiling. This analysis revealed four distinct prognostic molecular subtypes of PCNSL and developed algorithms for identification. Based on the multi-omics data classification, PCNSL was categorized into four clusters (CS). The CS1 and CS2 exhibited an immune-cold hypermethylated profile yet displayed distinct clinical behavior. The CS3 was characterized by meningeal infiltration and a high prevalence of HIST1H1E mutation; notably, only tumor cells from the CS3 were identified in the CSF in cases with meningeal infiltration, offering a potential diagnostic marker for PCNSL typing. The ‘immune-hot’ Cluster CS4 was enriched with mutations and exhibited increased JAK signal and NF-kB activity (34). These four molecular patterns, with their unique prognostic implications in PCNSL, lay the groundwork for future clinical stratification and the development of targeted interventions tailored to specific subtypes. Cluster CS3 may exhibit sensitivity to BTK inhibitors, while CS1 may derive therapeutic benefit from the loss of CDKN2A/B. The CS2 subtype potentially responds to the inhibition of IRF4, SPIB, MEIS1, and demethylation agents. Additionally, CS2 is characterized by elevated DNA methylation levels (34). Stratification based on distinct metabolic profiles can inform treatment strategies and potentially enhance prognostic outcomes.

### 4.6 Cognitive function assessment

Cognitive function in patients with CNS tumors can be compromised by both the cancer itself and the neurotoxic effects of treatment, resulting in a diminished quality of life and impaired social competence. Therefore, it is imperative to conduct essential cognitive screening and assessment for these patients using the Mini-Mental Status Examination (MMSE), a validated screening tool for cognitive function (35).

## 5 Treatment

### 5.1 Standard treatment

#### 5.1.1 Induction therapy of PCNSL

NCCN and ESMO guidelines prioritize enrollment in clinical trials for patients newly diagnosed with PCNSL. For those who are not eligible for clinical trials, both NCCN and ESMO guidelines advocate for induction regiments incorporating high-dose methotrexate (HD-MTX), such as R-MT or R-MPV.

There are distinctions between the NCCN and ESMO guidelines regarding the recommended regimen and HD-MTX dosage (**Table 1**). The NCCN guidelines specify two points: 1. R-M/R-MT can serve as an induction regimen, requiring an MTX of 8g/m^2^; 2. If R-M is combined with additional chemotherapy agents (R-MPV/R-MT) and consolidation with whole brain radiotherapy (WBRT) consolidation is under consideration, the MTX dosage can be reduced to 3.5g/m^2^. In contrast, ESMO recommends R-M in combination with multiple chemotherapeutic agents for induction and sets the minimum MTX dosage at 3g/m^2^.

**Table 1:** Comparison of NCCN and ESMO guidelines.

	NCCN	ESMO
Induction		
common		
	R-MT^1^/R-MPV^2^/R-MATRix^3^	
different		
	R-M (MTX 8.0g/m^2^)	MATRix
		R ± MBVP^4^
		MPV
		(MTX ≥3.0g/m^2^)
Consolidate and maintenance		
common		
	WBRT/ASCT	
different		
	HD-AraC ± etoposide	Watchful waiting**
	TMZ (after WBRT)	Maintenance therapy(lenalidomide)
	R-M based regimen*	
	Best supportive care	
R/R		
common		
	Retreat with HD-MTX	
	HD-AraC/Lenalidomide/Ibrutinib/TMZ	
different		
	Systemic and/or intra-CSF	HD-ifosfamide-based chemotherapy
	Focal irradiation	HD-Arac-based chemotherapy
	Involved-field RT	
	Rituximab + TMZ	
	Lenalidomide + rituximab	
	Pemetrexed	
	Pomalidomide	
	R-MBVP	

*Continue monthly for up to 1 y; **Patients not suitable for ASCT after CR.

1. R-MT, Rituximab + HD-MTX + Temozolomide;

2. R-MPV, Rituximab + HD-MTX + Procarbazine + Vincristine;

3. R-MATRix, Rituximab + HD-MTX + HD-AraC+ Thiotepa;

4. R ± MBVP, Rituximab ± HD-MTX + Carmustine + Teniposide +Methylprednisolone.

Patients who are intolerant to HD-MTX may consider alternative chemotherapy regimens. WBRT is suggested for patients who are ineligible for chemotherapy due to advanced age, poor performance status, or significant debilitation. For PCNSL patients who cannot tolerate chemotherapy, the ESMO guidelines recommend palliative strategies, including corticosteroids, oral alkylating agents such as temozolomide, carmustine, and procarbazine, with or without rituximab, WBRT, BTK inhibitors, and immunomodulators. In addition to intraocular chemotherapy, the NCCN guidelines also consider orbital radiotherapy (RT) for PCNSL patients exhibiting vitreoretinal involvement on ophthalmic examination who show no response to systemic therapy. The NCCN guidelines further recommend intra-CSF systemic treatment for patients with positive CSF findings who cannot tolerate systemic chemotherapy. Moreover, for patients with positive CSF or spinal MRI, the NCCN guidelines advocate focal spinal RT. Regarding intrathecal injection, the ESMO guidelines deem it appropriate for patients who are CSF-positive but cannot tolerate chemotherapy as a first-line treatment for those with persistent meningeal disease after initial CSF positivity.

#### 5.1.2 Consolidation therapy of PCNSL

Patients who achieve complete remission (CR), partial remission (PR), or stable disease (SD) following induction therapy have options for consolidation therapy, which may include WBRT or autologous stem cell transplantation (ASCT). **Table 1** delineates the distinctions between NCCN and ESMO guidelines regarding consolidation therapy approaches. The NCCN guidelines stipulate that ASCT should be considered only for patients who have attained CR or unconfirmed CR (uCR). In contrast, ESMO views ASCT as a viable option for patients who have not experienced disease progression and are otherwise eligible. For patients not eligible for ASCT post-CR, the ESMO guidelines suggest watchful waiting, WBRT, or lenalidomide maintenance therapy, given the intensive combination chemotherapy induction regimen typically utilized. Clinical trials are currently underway to assess lenalidomide and procarbazine as maintenance therapy in the R-MP regimen. Conversely, the NCCN guidelines do not endorse a wait-and-see approach; instead, they recommend a monthly MTX regimen (3.5g/m^2^ ~ 8g/m^2^) with or without R or R/Temozolomide (TMZ) as standalone options.

#### 5.1.3 Recurrent/refractory PCNSL

NCCN and ESMO guidelines prioritize enrollment in clinical trials for both R/R PCNSL and newly treated PCNSL. HD-MTX reinduction is acknowledged as an effective strategy for patients experiencing advanced recurrence, defined as ≥12 months post-HD-MTX-based chemotherapy by NCCN. For patients with early recurrence occurring within < 12 months or those with refractory treatment, alternative non-MTX regiments, such as HD-AraC chemotherapy, are recommended by the NCCN. The NCCN and ESMO guidelines endorse ibrutinib, TMZ, or lenalidomide for treating R/R PCNSL. Additionally, ESMO advocates for HD-ifosfamide-based chemotherapy. In contrast, the NCCN tailors its regimen recommendations to various conditions, including prior WBRT, prior HD-MTX-based regimen without prior RT, and ASCT, as detailed in **Table 1**.

#### 5.1.4 PTL

The CSCO guidelines utilize the 2014 Lugano staging criteria for the stratification of PTL. The preferred treatment for PTL, according to these guidelines, is radical orchiectomy followed by 6 to 8 cycles of R-CHOP and prophylactic RT (25 to 30Gy) to the contralateral scrotum. When orchiectomy is not feasible, involved site radiotherapy becomes an alternative treatment approach. Given the high propensity for CNS relapse in PTL, CNS prophylaxis is also recommended. This includes intrathecal injections of methotrexate, with or without cytarabine, and two cycles of HD-MTX. A distinction is made between stages III and IV and stages and IIE, where DLBCL-like therapy is favored. For R/R PTL, clinical trials should be considered the first-line option. For those who do not meet the eligibility criteria for clinical trials, DLBCL-like therapy, Bruton’s tyrosine kinase inhibitors (BTKi), lenalidomide, Programmed cell death receptor 1 (PD-1) monoclonal antibody, chimeric antigen receptor T-cell therapy (CAR-T), and ASCT are viable treatment options.

#### 5.1.5 PVRL

The ESMO guidelines advocate for a treatment approach for PVRL that mirrors the regimen used for PCNSL, complemented by intravitreal injection of MTX to expedite the remission of intraocular disease. Patients who attain CR or PR may benefit from further consolidation with orbital radiotherapy. For patients who are intolerant to chemotherapy, oral temozolomide in conjunction with orbital radiotherapy or intravitreal MTX injection is recommended. For R/R PVRL, treatment options include R-DHAP (Rituximab-Dexamethasone-Cytarabine-Cisplatin) or R-ICE(Rituximab-Ifosfamide-Carboplatine-Toposide) regimens, followed by thiotepa-based consolidation with HDC-ASCT therapy. For patients with R/R PVRL who are generally in poor condition, alternative treatment options include topical therapy, ibrutinib, lenalidomide, or temozolomide.

### 5.2 Targeted drug

BTKi, immune checkpoint inhibitors, immunomodulatory agents, CD20 monoclonal antibodies, and CAR-T cell therapies targeting CD19 have been proven to have significant anti-tumor effects, with their blood-brain barrier penetration rates shown in **Table 2**.

**Table 2:** BBB penetration rate of the targeted drug.

Representative drug	Target	BBB penetration rate(%)
Zanubrutinib	BTK	2.39 ± 1.71^a^(people^1^) (61) 3.58^b^(SD rat) (62)
Tirabrutinib		8.5^b^(SD rat) (62)
Nivolumab	PD-1	0.88-1.9^c^(people) (63)
Lenalidomide	Ikaros	1.3-2.4^c^(people) (64)
Pomalidomide	Aiolos	39^d^(CD-IGS rat) (65)
Rituximab	CD20	0.1^c^(people) (40)
Tisagenlecleucel	CD19	–

^1^Zanubrutinib protein binding rates were as high as 94%, and the adjusted BBB penetration ratio was 42.7% ± 27.7%.

^a^Penetration rate (%) =C median, CSF/C median, plasma.

^b^Penetration rate (%) =C max, brain/C max, plasma.

^c^Penetration rate (%) =C CSF/C plasma.

^d^Penetration rate (%) =AUC ratio (Brain: Blood).

**Figure 3:**
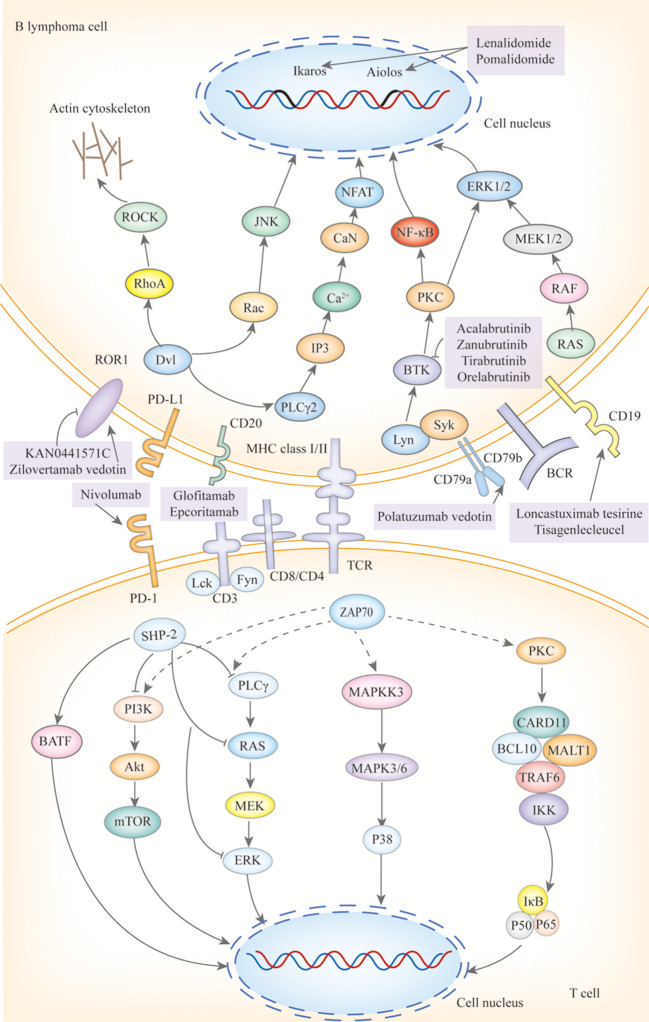
The mechanisms of action of targeted drugs and potential targeted drugs in IP-LBCL. ROR1, Receptor Tyrosine Kinase-Like Orphan Receptor 1; BCR, B-Cell Receptor; Dvl, Dishevelled; RhoA, Ras Homolog Family Member A; ROCK, Rho-Associated Coiled-Coil Kinase; Rac, Ras-Related C3 Botulinum Toxin Substrate; JNK, C-Jun N-Terminal Kinase; GSK3β, Glycogen Synthase Kinase 3 β; PI3K, Phosphatidylinositol3 kinase; Akt, Protein Kinase B; CaN, Calcineurin; PIP2, Phosphatidylinositol 4,5-Bisphosphate 3-Kinase; IP3:Inositol Triphosphate; NFAT; Nuclear Factor of Activated T Cells; Lyn, Tyrosine-Protein Kinase Lyn; Syk, Spleen Tyrosine Kinas; BTK, Bruton tyrosine kinase; PLCγ, Protein-Activated Phosphatidylinositol-Specific Phospholipase Cγ; BLNK1, B-Cell Linker Protein 1; VAV, Vav Guanine Nucleotide Exchange Factor; GRB2, Growth Factor Receptor-Bound Protein 2; SOS, Guanylate Release Factor; RAS, Rat Sarcoma; RAF, Rapidly Accelerated Fibrosarcoma; MEK1/2, Mitogen-Activated Protein Kinase Kinase 1 and 2; ERK1/2, Extracellular Regulated Protein Kinases; DAG, diglyceride; PKC, Protein Kinase C; CARD11, Caspase Recruitment Domain Containing Protein 11; BCL10, B-Cell Lymphoma 10 Protein; MALT1, Mucosa-Associated Lymphoid Tissue Lymphoma 1; TRAF, Tumor Necrosis Factor Receptor-Associated Factor; TAB2, Transforming Growth Factor-β Activated Kinase 1 Binding Protein 2; TAK1, Transforming Growth Factor Beta-Activated Kinase 1; IκB, Inhibitor of Kappa B; IKK, IκB Kinase; P50/65, NF-κB Subunits p50/p65; P100/52, NF-Kappa B Subunits p100/p52; TLR, Toll-Like Receptor; TRAM, Recombinant Translocation Associated Membrane Protein 1; MYD88, Myeloiddifferentiationfactor; IRAK, Interleukin 1 Receptor Associated Kinase; IAP, Inhibitor of Apoptosis Protein; NIK, NF-κB Inducing Kinase; JAK, Janus Kinase; STAT, Signal Transducer and Activator of Transcription; BAFF, B-cell Activating Factor of the TNF Family; IL-10R1, Interleukin-10 Receptor 1; CD, B-Cluster of Differentiation; Cluster of Differentiation 30/40; TNFR, Tumor Necrosis Factor Receptor; C-myc, Myelocellular Leukemia Oncogene; CyclinD1, Cyclin Dependent Kinase 1; PTEN, Phosphatase and Tensin Homolog; PD-L1, Programmed Cell Death Receptor 1; mTOR, MammalianTarget of Rapamycin; CIITA, MHC class II transactivator; β2M, β2-Microglobulin; ZAP70, Zeta-Chain Associated Protein Kinase 70; TCR, T-Cell Receptor; Lck, Lymphocyte-Specific Protein Tyrosine Kinase; Fyn, Src family tyrosine kinase; SHP-2, SH2 domain-containing protein-tyrosine phosphatase-2; MAPKK, Mitogen-Ativated Protein Kinase Kinase; MAPK, Mitogen-Activated Protein Kinase.

BTKi are a class of small molecules capable of traversing physiological barriers to specifically bind and inhibit the activity of BTK, thereby blocking downstream signal transduction pathways (**Figure 3**), including the NF-κB pathways. Among them, the My-T-BCR is a site for NF-κB activation and is extremely sensitive to BTK inhibition (possibly due to the autophagy of mutated MYD88 by BTK inhibitors), especially when there are concurrent mutations in MYD88 and CD79B, where the benefit of combining BTK inhibitors with chemotherapy is maximized. First-generation BTKi exhibit specific off-target inhibition effects, which has led to the preferential use of second-generation BTKi. Apart from acalabrutinib, which is currently under clinical investigation, both zanubrutinib and tirabrutinib have demonstrated efficacy and safety in PCNSL and PVRL, as shown in **Table 3**. Orelabrutinib has shown efficacy only in PCNSL. There are no reports on the application of orelabrutinib in PVRL or the use of BTK inhibitors in PTL. The programmed cell death receptor 1 (PD-1) and its ligand (primarily PD-L1) are expressed by tumor and microenvironmental cells. They mediate immune evasion of tumors by dampening the PTEN-PI3K-Akt and RAS-MEK-ERK signaling pathways and upregulating the expression of transcription factors such as Basic Leucine Zipper Transcriptional Factor ATF-like (BATF) (36), as shown in **Figure 3**. Drugs targeting PD-1/PD-L1 rejuvenate exhausted T cells in the TME. Apart from PVRL, both PCNSL and PTL have shown clinical and radiological responses following PD-1 blockade. A clinical trial investigated the effectiveness of the anti-PD-1 antibody Nivolumab in four patients with R/R PCNSL and one patient with PTL CNS relapse. The results indicated that all five patients exhibited clinical and radiographic responses to PD-1 blockade (37).

**Table 3:** Clinical Trials of Targeted Therapies for IP-LBCL.

Study	Time	Regimen	Median follow-up	Patients (n)	ORR (%)	CR (%)	OS	PFS
Bai et al. (66)	2024	BTKi combination therapy	28.7m**	26	96.2	76.9	NR* (median)	NR* (median)
Yang et al. (67)	2022	Orelabrutinib lenalidomide immunochemo- therapy	219d***	15	86.7	73.3	NR* (median)	9.8m** (median)
Maet al. (68)	2022	Pemetrexed lenalidomide	18m**	38	68.4	39.5	18m** (median)	6m** (median)
Yonezawa et al. (69)	2024	Tirabrutinib	37.1m**	44	63.6	20.5	NR* (median)	2.9m** (median)
Fox et al. (70)	2021	Thiotepa ifosfamide etoposide rituximab	21m**	27	52.0	14.8	5m**	3m** (median)
Han et al. (71)	2018	pomalidomide dexamethasone	16.5m**	25	48	20.7	-	5.3m**
Ghesquieres et al. (72)	2019	Lenalidomide rituximab	19.2m**	45	35.6	28.9	17.7m**	7.8m**

*NR: not reached; **m: months; ***d: days.

Immune modulators lenalidomide and pomalidomide exert their effects by binding to the cereblon protein within the E3 ubiquitin ligase complex, leading to the ubiquitination and degradation of specific proteins such as Ikaros and Aiolos, as shown in **Figure 3**. The degradation of these proteins plays a crucial role in regulating immunity and tumor cell growth. Additionally, lenalidomide has the ability to cross the blood-brain barrier and exhibits single-agent activity (38). Pomalidomide is 10 times more potent than lenalidomide, yet there are no clinical studies to assess its ability to cross the blood-brain barrier. However, research has shown that after a single oral administration of ^14^C-labeled pomalidomide to rats, radioactivity can be detected in the spinal cord and brain 3 hours later (39). Lenalidomide has demonstrated clinical activity in PCNSL, PTL, and PVRL, while pomalidomide has shown efficacy only in PCNSL and PVRL, as reported in **Table 3**. Its efficacy in PTL remains to be investigated.

The monoclonal antibody targeting CD20 (see **Figure 3**), rituximab, has been incorporated into guidelines. Although only 0.1% can cross the BBB (**Table 2**) (40), it plays a positive role in IP-LBCL (**Table 3**).

CD19-targeting CAR-T cell therapy (**Figure 3**) offers a novel option for IP-LBCL patients, introducing a new treatment paradigm that demonstrates unprecedented efficacy and significantly improves the prognosis of IP-LBCL patients. It has been reported that CD19 CAR-T cells can cross the BBB, but the extent of BBB penetration requires further investigation. A retrospective study leveraging the LOC network database demonstrated significantly improved clinical outcomes in the CAR-T cell therapy cohort relative to controls, with median progression-free survival (PFS) of 3 months versus not reported (NR) in controls (p < 0.001), and median overall survival (OS) of 4.7 months versus NR. Of the 25 CAR-T recipients, 23 (92%) developed cytokine release syndrome (CRS), while neurotoxicity events occurred in 17 patients (68%), 5 of whom (20%) experienced grade 3 or higher adverse events (41). A meta-analysis evaluating the toxicity and efficacy of CAR-T cell therapy in PCNSL revealed that 70% of PCNSL patients developed cytokine release syndrome (CRS) of any grade, including 13% with grade 3-4 severity, while 53% experienced immune effector cell-associated neurotoxicity syndrome (ICANS), with 18% classified as grade 3-4. Clinically, 56% of patients achieved complete remission (CR) and 37% maintained remission at 6 months. These findings suggest that the toxicity profile of anti-CD19 CAR-T cell therapy in PCNSL aligns with that reported in systemic diffuse large B-cell lymphoma (DLBCL), without evidence of elevated neurotoxicity. Furthermore, the therapy demonstrated robust efficacy with high rates of durable responses, supporting its safety and effectiveness in this patient population (42). Additionally, some patients experience disease progression or lack of response to CAR-T cells during infusion, relapse, or succumb to severe adverse reactions post-infusion. Enhancing the persistence of CAR-T cells *in vivo*, increasing their tumor cell-killing capacity, and reducing CRS and ICANS are current challenges that need to be addressed. There is an urgent need to develop new CAR-T products to overcome these limitations. Some clinical trials on CAR-T are detailed in **Table 4**.

**Table 4:** The clinical trials of CAR-T.

Study	Time	Median follow-up	Patients (n)	ORR (%)	CR (%)	OS	PFS	CRS (%)	CRS≥3 (%)	Median CRS onset	ICANS (%)	ICANS≥3 (%)	Median ICANS onset
Yu et al. (73)	2024	316d	22	90.9	68.2	NR^***^	NR	72.7	4.5	4d	36.4	4.5	8d
Mercadal et al. (74)	2024	26m	24	61	48	–	–	66.7	0	3.5d	33	8	6d
Frigault et al. (75)	2022	12.2m^*^	12	58.3	50	–	–	58.3	0	4d**	50	8	5d
Choquet et al. (41)	2024	20.8m	25	56	8	21.2m (median)	8.4m (median)	92	–	1d	68	20	1d
Karschnia et al. (76)	2023	12m	18	39	33	–	3.5m (median)	–	–	–	44.4	16.7	2.5d
Lacan et al. (77)	2023	12m	13	38	31	20m (median)	3m (median)	–	–	–	–	–	–

*m: months; **d: days; ***NR: not reached.

### 5.3 Potential targeted drugs

Small molecule inhibitors of ROR1, bispecific antibodies, antibody-drug conjugates (ADCs), and carriers for large molecular substances have not yet been applied to IP-LBCL, but all show great clinical potential.

ROR1 small molecule inhibitors, such as KAN0441571C (**Figure 3**), target ATP-binding sites within the ROR1 TK domain (43) and down-regulate ROR1 signaling pathways, including non-classical Wnt pathways (PKC and PI3Kδ/AKT/mTOR) and classical Wnt/β-catenin pathways, which have been shown to induce apoptosis of ROR1-positive DLBCL cell lines. The high ROR1 expression in IP-LBCL suggests its potential as a novel therapeutic target for IP-LBCL (11). Further research in this area is ongoing.

Bispecific antibodies (**Figure 3**) bridge CD3 on T cells and tumor antigens. They guide T cells to the tumor site to form an immunological synapse, triggering a cascade reaction of perforin, granzyme, and cytokine release, thereby lysing tumor cells. This reaction is independent of the interaction between MHC and T cells (43). Therefore, bispecific antibodies may still have a cytotoxic effect for IP-LBCL, which often lacks MHC and causes immune escape. The CD20×CD3 bispecific antibody glofitamab can partially penetrate the blood-brain barrier. Although the average concentration in cerebrospinal fluid is only 0.1-0.4% (40) of that in peripheral blood, it is sufficient to induce clinical and radiological responses in patients with PCNSL. Godfrey and colleagues enrolled four patients with DLBCL involving the central nervous system and administered glofitamab (median number of cycles, 6.5). Glofitamab was detected in the CSF of all four patients, with concentrations ranging from 0.00632 to 0.0296 g/mL. Moreover, CSF samples from three patients significantly induced T-cell activation (upregulation of CD25 and CD69), enhancing T-cell cytotoxicity (40).

ADCs, such as loncastuximab tesirine (an anti-CD19 antibody conjugated with a potent pyrrolobenzodiazepine), zilovertamab vedotin (an anti-ROR1 antibody conjugated with monomethyl auristatin E (MMAE)), and polatuzumab vedotin (an anti-CD79 antibody conjugated with MMAE), represent an innovative therapeutic approach that combines the targeting capability of monoclonal antibodies with the potent cytotoxicity of small molecule drugs. These ADCs induce apoptosis through four processes: targeted binding (**Figure 3**), internalization, toxin release, and cell killing. Currently, no ADCs have been specifically studied for IP-LBCL. IP-LBCL is a rare B-cell lymphoma with similar biological characteristics to DLBCL so that ADCs may have potential therapeutic value. Efficacy and safety data from DLBCL can provide a reference for treating IP-LBCL.

Penetrating the BBB to cross physiological barriers and enter immune-privileged sites is challenging in developing immunotherapeutic drugs for IP-LBCL. Carriers such as the transferrin receptor (TfR), CD98hc, and gold nanoparticles (AuNPs) (44) that form tight junctions have paved the way for significant molecular substances to enter these privileged areas. TfR and CD98hc are molecules expressed by brain vascular endothelium, and animal experiments have proven that TfR and CD98hc can promote the transcytosis of significant molecular substances across the BBB (45). AuNPs can temporarily increase BBB permeability through local biophysical effects generated by their interaction with laser pulses. Monoclonal antibodies, bispecific antibodies, and even ADCs in combination with TfR, CD98hc, or AuNPs seem to offer a solution to the challenge of drug penetration through the blood-brain barrier.

## 6 Prognosis

For PCNSL, the current recommended prognostic scoring systems are derived from the International Extranodal Lymphoma Working Group (IELSG) and the Model for Memorial Sloan-Kettering Cancer Centre (MSKCC). However, these models have their limitations. In practical clinical settings, the IELSG score may be challenging to apply for some patients due to the contraindications of lumbar puncture or the unavailability of lactate dehydrogenase (LDH) data, hindering practical prognosis evaluation. The MSKCC risk stratification, which relies solely on age and Karnofsky Performance Status (KPS), may be biased, leading to potentially inaccurate prediction outcomes. Consequently, modifications have been made to the IELSG and MSKCC scoring models. New prognostic factors have been incorporated, including 18-month progression of disease (POD18), tumor-associated macrophages, LDH levels, and lymphocyte ratios. The combination of POD18 with an improved IELSG score (age≥54 years, ECOG≥2, deep brain structure involvement, elevated CSF protein, LDH ratios above the upper limits of normal > 0.75) demonstrated robust discriminability (C-index=0.828), significantly outperforming the standard IELSG score (C-index=0.625). Similarly, the AUC for this combined model was higher than that of the IELSG (46). To validate the prognostic significance of combined tumor-associated macrophage (TAMs) related biomarkers, the expression PD-1 on TAMs and the ratio of alternatively activated macrophage (M2) to classically activated macrophages (M1) were integrated into the IELSG, resulting in the IELSG-M index. Kaplan-Meier plots indicated that the IESLG-M index more effectively stratified patients into low-, intermediate-, and high-risk prognosis subgroups than the IELSG. The areas under the receiver operating characteristic curves for IELSG-M were 0.844 for OS, surpassing the IELSG model’s 0.580 (47). Furthermore, studies have revealed that while there is a significant difference in the OS rates among low-, intermediate-, and high-risk groups according to the MSKCC scoring system, the 5-year OS rate is 33.3%, 40.9% and 11.6%, respectively, the validation cohort’s low - and intermediate-risk groups could not be distinguished. The novel PCNSL lactate-dehydrogenase-to-lymphocyte ratio (LLR) scoring system successfully differentiated patients in the validation cohort into low-, intermediate- and high-risk groups, with 5-year OS rates of 61.9%, 30.2%, and 11.6%, respectively (48).

Researchers have proposed several new prognostic models for PCNSL, including the Xijing model, the two-factor prediction model, the Taipei score, and the NNI-NCCS score (**Table 5**). The Xijing model incorporates four variables: number of lesions, β2-microglobulin (β2-MG), systemic inflammatory response index (SIRI), and KPS. Patient scores are calculated by assigning different points to these variables. A cut-off value is determined to stratify the risk. When applied to a cohort study of 72 PCNSL patients, the Xijing model demonstrated that the median OS was 48, 19, and 5 months in the low-, medium-, and high-risk groups, respectively, and the median PFS was 10, 5.5, and 3 months, respectively. These values were shorter than those obtained through IELSG and MSKCC score stratification, suggesting that the Xijing model may offer more refined prognostic stratification (49). The two-factor prognostic model is based on Eastern Cooperative Oncology Group (ECOG) PS and albumin levels (ECOG PS >1 and albumin ≤4.1), with each parameter assigned a point, categorizing newly diagnosed PCNSL patients into three risk groups (Low-risk group: 0 points; Intermediate-risk group: 1 point; High-risk group: 2 points). The 5-year survival rates for these groups were 47.5%, 36.9%, and 11.9%, respectively, showing significant OS differences. Unlike the MSKCC scoring system, which showed no statistical difference in OS between the low- and intermediate-risk groups, this two-factor model provides a simple yet effective prognostic tool for newly diagnosed PCNSL patients (50). The Taipei score is based on three risk factors: age≥80, deep brain lesions, and ECOG≥2, with each factor assigned one point. The model was used to evaluate 101 newly diagnosed PCNSL patients, with median PFS in the low-, intermediate, high-, and very high-risk groups being 3.9, 1.7, 0.7, and 0.1 years, respectively. The higher the score, the worse the OS (51). The Taipei score retains factors with prognostic value in assessing OS and PFS: Age and deep brain lesions, with 80 years as the age dividing line (50 years for MSKCC and 60 years for IELSG), and significantly differentiates the validation risk group while providing prognostic stratification for PFS and OS compared to the IELSG and MSKCC. A novel PCNSL prognostic risk-scoring system has been proposed based on KPS and six mutant genes (BRD4, EBF1, BTG1, CCND3, STAG2, and TMSB4X). Using a calculated cut-off value, patients are divided into low-risk (≤0.504) and high-risk (>0.504) groups, with the high-risk group showing significantly lower OS (52). This system enriches the understanding of the genetic mechanisms of PCNSL and offers a new method for assessing the prognostic risk. In the rituximab era, the MSKCC had a poor concordance index of 0.57. The NCCS-NNI score classifies patients into low-, medium-, and high-risk groups based on age and post-steroid neutrophil-lymphocyte ratio (NLR) and pre-steroid NLR. The 2-year mortality rates were 5%, 38% i, and 73% for the low-, medium-, and high-risk groups, respectively (53). An increase in post-steroid NLR may serve as a biomarker for prolonged survival and a lower rate of progression after chemotherapy initiation. The NCCS-NNI score is more discriminating and better calibrated in internal validation than MSKCC and aligns more closely with modern healthcare and treatment options. Furthermore, by integrating genome-wide data from multiple omics, researchers have identified four molecular patterns in PCNSL with unique prognostic effects labeled CS1-CS4. OS was significantly different among these groups, with patients in CS4 having the most prolonged survival, substantially longer than those with CS2 and CS3, and slightly longer than those with CS1. These differences remained significant after adjusting for age and KPS. CS4 was also independently relevant when considering PFS (34). This provides a basis for future clinical stratification and targeted interventions based on subtypes.

**Table 5:** New prognostic model of PCNSL.

Model	Factors	Risk stratification	Prognostic value	
			median OS	median PFS
**IESLG (** 49)	Age LDH ECOG CSF protein Deep brain structure	Low-risk	89m*	18m
		Medium-risk	23m	7m
		High-risk	6m	4m
**MSKCC (** 49)	Age KPS	Low-risk	NR**	8.5m
		Medium-risk	23m	8.5m
		High-risk	12m	4.5m
**POD18-IELSG (** 46)	POD18 Age Ratios to upper limits of normal for LDH ECOG CSF protein Deep brain structure	Low-risk	–	–
		Intermediate‐risk		-
		High‐risk		–
**IELSG‐M index (** 47)	PD-1 expression on TAMs M2/M1 Age LDH ECOG CSF protein Deep brain structure	Low‐risk	NR	NR
		Intermediate‐risk	29.9m	29.57m
		High‐risk	11.7m	11.57m
**The novel PCNSL LLR scoring system** (34)	LLR Age KPS	Low-risk	74m	–
		Intermediate-risk	33m	-
		High-risk	17m	–
**The Xijing model (** 49)	Lesion number β2-MG SIRI KPS	Low-risk	48m	10m
		Medium-risk	19m	5.5m
		High-risk	5m	3m
**The two−factor prognostic model (** 50)	ECOG PS Albumin	Low-risk	55m	–
		Intermediate-risk	46m	–
		High-risk	23m	–
**The Taipei Score (** 51)	Age ECOG Deep brain lesions	Low-risk	NR	3.9y***
		Intermediate-risk	3.1y	1.7y
		High-risk	NR	0.7y
		Very high-risk	NR	0.1y
**The prognostic risk scoring system (** 52)	KPS Six mutated genes (BRD4, EBF1, BTG1, CCND3, STAG2, and TMSB4X)	Low-risk	-	-
		High-risk	–	–
**The NCCS-NNI score (** 53)	Age Pre-steroids NLR Post-steroids NLR	Low-risk	-	-
		Medium-risk	–	–
		High-risk	-	-
**Multi-omic grouping (** 34)	Immune state	CS1	–	–
		CS2	-	-
		CS3	–	–
		CS4	-	-

*m: months; **NR: not reached; ***y: years.

The International Prognostic Index (IPI) is limited in its ability to stratify the prognostic of patients with PTL, necessitating the development of a more refined prognostic model. Current research has identified several factors associated with poor prognosis in PTL patients: the presence of B symptoms, specific genetic alterations such as BTG2 mutation (54), TP53 mutation, HLA deletion, a serum LDH to CSF LDH ratio of6.5 or higher (55), loss of 5-hydroxymethylcytosine (56), low T lymphocyte content or low expression of T lymphocyte characteristic genes in TME, and p53 immunohistochemical overexpression (defined as positive immunohistochemical staining of at least 50% of tumor cells in testicular tissue (57)). A molecular analysis of 25 PTL patients revealed that those with HLA mutations had a significantly worse prognosis than those with TP53 mutations (58). Leivonen et al. categorized patients into high, medium, and low groups based on the expression of 121 T lymphocyte characteristic genes. Cox multivariate analysis showed that the low expression of these genes was an independent prognostic factor, significantly correlating with poor PFS, OS, and disease-specific survival (DSS), with hazard ratios of 2.810, 3.267, and 2.910, respectively (59).

Research on the prognosis of PVRL is limited. However, one study suggested that patients with lower serum IgA levels had a higher relapse rate and lower survival rates compared to those with higher IgA levels (60).

## 7 Outlook

Recent research advancements have provided new perspectives for the diagnosis and treatment of IP-LBCL. The integration of genomics, proteomics, metabolomics, immunology, and radiomics with clinical features is anticipated to facilitate the development of novel, specific predictive and diagnostic biomarkers. Utilizing AI to develop new prognostic models will aid in guiding clinical decision-making.

Exploring potential therapeutic targets such as ROR1 provides direction for the development of new targeted drugs; designing and developing new antibody-based drugs based on the distinct antigen expression profiles that may exist in different types of IP-LBCL; and facilitating the transport of large molecular drugs across the BBB using carriers like TfR, CD98hc, and exosomes, or by optimizing drug molecular structures and administration methods, can all increase drug concentration and activity in immune-privileged sites. Further optimizing the structural design of CAR-T cells to enhance their targeting and persistence in immune-privileged areas and developing dual-target and multi-target CAR-T cells to overcome drug resistance and broaden the therapeutic spectrum will offer more effective treatment options for IP-LBCL patients, significantly improving their survival rates.

By combining various treatment modalities such as targeted therapy, immune checkpoint inhibitors, CAR-T cell therapy, and antibody-based drugs in a rationally personalized manner according to the genetic, protein, and immune microenvironmental characteristics of IP-LBCL patients, the combination of BTK inhibitors with PD-1/PD-L1 inhibitors may synergistically overcome tumor immune escape, enhancing the efficacy and reducing adverse reactions in IP-LBCL patients.

Given the rarity of the other three types of IP-LBCL, a comprehensive and systematic summary of these subtypes is lacking, which represents a limitation of this review.

In summary, breaking the immune escape of IP-LBCL and developing effective therapeutic strategies will pave new avenues and demonstrate a promising future for the treatment of IP-LBCL!

## Supplementary material

Supplementary Table 1 Pathological, molecular, and genetic changes of IP-LBCL. *DLBCL accounted for 95% of PCNSL, and the remaining pathological types accounted for 5%. **DLBCL accounted for 80-90% of PCNSL, and the remaining pathological types accounted for 10-20%.

## References

[1] Roschewski M Phelan JD Jaffe ES. Primary large B-cell lymphomas of immune-privileged sites. Blood. (2024) 144(25):2593–603. doi: 10.1182/blood.2023020911 PMC11862818 38635786

[2] Bairey O Lebel E Buxbaum C Porges T Taliansky A Gurion R. A retrospective study of 222 patients with newly diagnosed primary central nervous system lymphoma-outcomes indicative for improved survival overtime. Hematol Oncol. (2023) 41:838–47. doi: 10.1002/hon.3198 37403752

[3] Ferreri AJM Calimeri T Cwynarski K Dietrich J Grommes C Hoang-Xuan K. Primary central nervous system lymphoma. Nat Rev Dis Primers. (2023) 9:29. doi: 10.1038/s41572-023-00439-0 37322012 PMC10637780

[4] Liu KT Chang YC Lin YC Chang JL. Unusually aggressive primary testicular diffuse large B-cell lymphoma initially presenting as systemic disseminating metastases in older adult men: A case report. Ann Med Surg (2012). (2023) 85:4106–11. doi: 10.1097/ms9.0000000000001018 PMC10406097 37554871

[5] Shah S Sreenivasan S Kancharla P Khan C Samhouri Y. Primary testicular lymphoma: single center experience. Cancer Diagn Prognosis. (2023) 3:139–44. doi: 10.21873/cdp.10192 PMC9949543 36875297

[6] Soussain C Malaise D Cassoux N. Primary vitreoretinal lymphoma: A diagnostic and management challenge. Blood. (2021) 138:1519–34. doi: 10.1182/blood.2020008235 34036310

[7] Petrova TV Koh GY. Biological functions of lymphatic vessels. Sci (New York N.Y.). (2020) 369(6500):eaax4063. doi: 10.1126/science.aax4063 32646971

[8] Kumaria A. Insights in primary central nervous system lymphoma: A role for glymphatics? Brain Tumor Pathol. (2021) 38:290–91. doi: 10.1007/s10014-021-00414-1 34532755

[9] Radke J Ishaque N Koll R Gu Z Schumann E Sieverling L. The genomic and transcriptional landscape of primary central nervous system lymphoma. Nat Commun. (2022) 13:2558. doi: 10.1038/s41467-022-30050-y 35538064 PMC9091224

[10] Pollari M Leivonen SK Leppä S. Testicular diffuse large B-cell lymphoma-clinical, molecular, and immunological features. Cancers. (2021) 13(16):4049. doi: 10.3390/cancers13164049 34439203 PMC8392512

[11] Ghaderi A Daneshmanesh AH Moshfegh A Kokhaei P Vågberg J Schultz J. Ror1 is expressed in diffuse large B-cell lymphoma (Dlbcl) and a small molecule inhibitor of ror1 (Kan0441571c) induced apoptosis of lymphoma cells. Biomedicines. (2020) 8(6):170. doi: 10.3390/biomedicines8060170 32586008 PMC7344684

[12] Minderman M Amir A Kraan W Schilder-Tol EJM Oud M Scheepstra CG. Immune evasion in primary testicular and central nervous system lymphomas: hla loss rather than 9p24.1/pd-L1/pd-L2 alterations. Blood. (2021) 138:1194–97. doi: 10.1182/blood.2021011366 PMC9632757 34125179

[13] Twa DDW Mottok A Savage KJ Steidl C. The pathobiology of primary testicular diffuse large B-cell lymphoma: implications for novel therapies. Blood Rev. (2018) 32:249–55. doi: 10.1016/j.blre.2017.12.001 29289361

[14] Bonzheim I Sander P Salmerón-Villalobos J Süsskind D Szurman P Gekeler F. The molecular hallmarks of primary and secondary vitreoretinal lymphoma. Blood Adv. (2022) 6:1598–607. doi: 10.1182/bloodadvances.2021004212 PMC8905692 34448823

[15] Li J Tang X Luo X Liu L Li D Yang L. Clinicopathological analysis and specific discriminating markers of interleukin detection in cerebrospinal fluid with primary central nervous system lymphoma: results from a retrospective study. Ann Hematol. (2023) 102:2153–63. doi: 10.1007/s00277-023-05301-7 37289220

[16] Sun X Lv L Wu Y Cui Q Sun S Ji N. Challenges in the management of primary central nervous system lymphoma. Crit Rev Oncology/hematol. (2023) 188:104042. doi: 10.1016/j.critrevonc.2023.104042 37277008

[17] Kuo DE Wei MM Knickelbein JE Armbrust KR Yeung IYL Lee AY. Logistic regression classification of primary vitreoretinal lymphoma versus uveitis by interleukin 6 and interleukin 10 levels. Ophthalmology. (2020) 127:956–62. doi: 10.1016/j.ophtha.2020.01.042 PMC7311235 32197914

[18] Huang RS Mihalache A Popovic MM Cruz-Pimentel M Pandya BU Muni RH. Diagnostic methods for primary vitreoretinal lymphoma: A systematic review. Survey Ophthalmol. (2024) 69:456–64. doi: 10.1016/j.survophthal.2023.12.001 38163550

[19] Ambady P Hu LS Politi LS Anzalone N Barajas RF Jr. Primary central nervous system lymphoma: advances in mri and pet imaging. Ann Lymphoma. (2021) 5:27. doi: 10.21037/aol-20-53 35994050 PMC9387672

[20] Han Y Wang ZJ Li WH Yang Y Zhang J Yang XB. Differentiation between primary central nervous system lymphoma and atypical glioblastoma based on mri morphological feature and signal intensity ratio: A retrospective multicenter study. Front Oncol. (2022) 12:811197. doi: 10.3389/fonc.2022.811197 35174088 PMC8841723

[21] Kumar Madaan P Jain P Sharma A Malik A Nair Misra R. Imaging of primary testicular lymphoma with unusual intraabdominal spread along the spermatic cord and gonadal vein. Radiol Case Rep. (2021) 16:419–24. doi: 10.1016/j.radcr.2020.12.003 PMC7750148 33363674

[22] Takase H Arai A Iwasaki Y Imai A Nagao T Kawagishi M. Challenges in the diagnosis and management of vitreoretinal lymphoma - clinical and basic approaches. Prog Retinal Eye Res. (2022) 90:101053. doi: 10.1016/j.preteyeres.2022.101053 35210172

[23] Melli B Gentile P Nicoli D Farnetti E Croci S Gozzi F. Primary vitreoretinal lymphoma: current diagnostic laboratory tests and new emerging molecular tools. Curr Oncol (Toronto Ont.). (2022) 29:6908–21. doi: 10.3390/curroncol29100543 PMC9600627 36290820

[24] Gupta T Manjali JJ Kannan S Purandare N Rangarajan V. Diagnostic performance of pretreatment 18f-fluorodeoxyglucose positron emission tomography with or without computed tomography in patients with primary central nervous system lymphoma: updated systematic review and diagnostic test accuracy meta-analyses. Clin Lymphoma Nyeloma Leukemia. (2021) 21:497–507. doi: 10.1016/j.clml.2021.03.011 33947632

[25] Hyun Suh C Kim HS Ahn SS Seong M Han K Park JE. Body ct and pet/ct detection of extracranial lymphoma in patients with newly diagnosed central nervous system lymphoma. Neuro-oncology. (2022) 24:482–91. doi: 10.1093/neuonc/noab234 PMC8917397 34611696

[26] Kim HO Kim JS Kim S-O Chae SY Oh SJ Seo M. Clinicopathological characteristics of primary central nervous system lymphoma with low 18f-fludeoxyglucose uptake on brain positron emission tomography. Medicine. (2020) 99(20):e20140. doi: 10.1097/md.0000000000020140 32443328 PMC7254841

[27] Rozenblum L Galanaud D Houillier C Soussain C Baptiste A Belin L. 18f]Fdg pet-mri provides survival biomarkers in primary central nervous system lymphoma in the elderly: an ancillary study from the blocage trial of the loc network. Eur J Nucl Med Mol Imaging. (2023) 50:3684–96. doi: 10.1007/s00259-023-06334-w 37462774

[28] Barajas RF Politi LS Anzalone N Schöder H Fox CP Boxerman JL. Consensus recommendations for mri and pet imaging of primary central nervous system lymphoma: guideline statement from the international primary cns lymphoma collaborative group (Ipcg). Neuro-oncology. (2021) 23:1056–71. doi: 10.1093/neuonc/noab020 PMC8248856 33560416

[29] Zhai Y Zhou X Wang X. Novel insights into the biomarkers and therapies for primary central nervous system lymphoma. Ther Adv Med Oncol. (2022) 14:17588359221093745. doi: 10.1177/17588359221093745 35558005 PMC9087239

[30] Bobillo S Crespo M Escudero L Mayor R Raheja P Carpio C. Cell free circulating tumor DNA in cerebrospinal fluid detects and monitors central nervous system involvement of B-cell lymphomas. Haematologica. (2021) 106:513–21. doi: 10.3324/haematol.2019.241208 PMC7849551 32079701

[31] Wang X Su W Gao Y Feng Y Wang X Chen X. A pilot study of the use of dynamic analysis of cell-free DNA from aqueous humor and vitreous fluid for the diagnosis and treatment monitoring of vitreoretinal lymphomas. Haematologica. (2022) 107:2154–62. doi: 10.3324/haematol.2021.279908 PMC9425330 35142151

[32] Zajdel M Rymkiewicz G Sromek M Cieslikowska M Swoboda P Kulinczak M. Tumor and cerebrospinal fluid micrornas in primary central nervous system lymphomas. Cancers. (2019) 11(11):1647. doi: 10.3390/cancers11111647 31731456 PMC6895823

[33] Minezaki T Usui Y Asakage M Takanashi M Shimizu H Nezu N. High-throughput microrna profiling of vitreoretinal lymphoma: vitreous and serum microrna profiles distinct from uveitis. J Clin Med. (2020) 9(6):1844. doi: 10.3390/jcm9061844 32545709 PMC7356511

[34] Hernández-Verdin I Kirasic E Wienand K Mokhtari K Eimer S Loiseau H. Molecular and clinical diversity in primary central nervous system lymphoma. Ann Oncol. (2023) 34:186–99. doi: 10.1016/j.annonc.2022.11.002 36402300

[35] Chen T Liu Y Wang Y Chang Q Wu J Wang Z. Evidence-based expert consensus on the management of primary central nervous system lymphoma in China. J Hematol Oncol. (2022) 15:136. doi: 10.1186/s13045-022-01356-7 36176002 PMC9524012

[36] Ai L Xu A Xu J. Roles of pd-1/pd-L1 pathway: signaling, cancer, and beyond. Adv Exp Med Biol. (2020) 1248:33–59. doi: 10.1007/978-981-15-3266-5_3 32185706

[37] Nayak L Iwamoto FM Lacasce A Mukundan S Roemer MGM Chapuy B. Pd-1 blockade with nivolumab in relapsed/refractory primary central nervous system and testicular lymphoma. Blood. (2017) 129:3071–73. doi: 10.1182/blood-2017-01-764209 PMC5766844 28356247

[38] Goldfinger M Xu M Bertino JR Cooper DL. Checking in on lenalidomide in diffuse large B cell lymphoma. Clin Lymphoma Nyeloma Leukemia. (2019) 19:e307–e11. doi: 10.1016/j.clml.2019.02.012 30926391

[39] Gozzetti A Cerase A. Novel agents in cns myeloma treatment. Cent Nervous Syst Agents Ned Chem. (2014) 14:23–7. doi: 10.2174/1871524914999140818111514 25134940

[40] Godfrey JK Gao L Shouse G Song JY Pak S Lee B. Glofitamab stimulates immune cell infiltration of cns tumors and induces clinical responses in secondary cns lymphoma. Blood. (2024) 144:457–61. doi: 10.1182/blood.2024024168 PMC11302446 38484137

[41] Choquet S Soussain C Azar N Morel V Metz C Ursu R. Car T-cell therapy induces a high rate of prolonged remission in relapsed primary cns lymphoma: real-life results of the loc network. Am J Hematol. (2024) 99:1240–49. doi: 10.1002/ajh.27316 38586986

[42] Cook MR Dorris CS Makambi KH Luo Y Munshi PN Donato M. Toxicity and efficacy of car T-cell therapy in primary and secondary cns lymphoma: A meta-analysis of 128 patients. Blood Adv. (2023) 7:32–9. doi: 10.1182/bloodadvances.2022008525 PMC9813524 36260735

[43] Li L Huang W Ren X Wang Z Ding K Zhao L. Unlocking the potential: advancements and future horizons in ror1-targeted cancer therapies. Sci China Life Sci. (2024) 67(12):2603–16. doi: 10.1007/s11427-024-2685-9 39145866

[44] Li X Vemireddy V Cai Q Xiong H Kang P Li X. Reversibly modulating the blood-brain barrier by laser stimulation of molecular-targeted nanoparticles. Nano Lett. (2021) 21:9805–15. doi: 10.1021/acs.nanolett.1c02996 PMC8616836 34516144

[45] Pornnoppadol G Bond LG Lucas MJ Zupancic JM Kuo YH Zhang B. Bispecific antibody shuttles targeting cd98hc mediate efficient and long-lived brain delivery of iggs. Cell Chem Biol. (2024) 31:361–72.e8. doi: 10.1016/j.chembiol.2023.09.008 37890480 PMC10922565

[46] Du KX Shen HR Pan BH Luthuli S Wang L Liang JH. Prognostic value of pod18 combined with improved ielsg in primary central nervous system lymphoma. Clin Trans Oncol. (2024) 26:720–31. doi: 10.1007/s12094-023-03292-5 37558851

[47] Sun X Wang C Chen C Huang J Wu X Wang Y. Combined tumor-associated macrophages biomarker predicting extremely poor outcome of patients with primary central nervous system lymphoma. Hematol Oncol. (2021) 39:625–38. doi: 10.1002/hon.2926 34543472

[48] Gao Y Wei L Kim SJ Wang L He Y Zheng Y. A novel prognostic marker for primary cns lymphoma: lactate dehydrogenase-to-lymphocyte ratio improves stratification of patients within the low and intermediate mskcc risk groups. Front Oncol. (2021) 11:696147. doi: 10.3389/fonc.2021.696147 34422649 PMC8370855

[49] Wu Z Wang C Lyu Y Lin Z Lu M Wang S. A novel inflammation-related prognostic model for predicting the overall survival of primary central nervous system lymphoma: A real-world data analysis. Front Oncol. (2023) 13:1104425. doi: 10.3389/fonc.2023.1104425 37056341 PMC10086228

[50] Wei L Gao Y Prochazka KT Liu R Wang L Liu B. A novel prognostic model based on pretreatment serum albumin and ecog ps for primary cns lymphoma: an international, multi-center study. J Neuro-oncol. (2023) 163:301–11. doi: 10.1007/s11060-023-04337-z 37231231

[51] Liu CJ Lin SY Yang CF Yeh CM Kuan AS Wang HY. A new prognostic score for disease progression and mortality in patients with newly diagnosed primary cns lymphoma. Cancer Med. (2020) 9:2134–45. doi: 10.1002/cam4.2872 PMC7064125 32011103

[52] Yuan X Yu T Zhao J Jiang H Hao Y Lei W. Analysis of the genomic landscape of primary central nervous system lymphoma using whole-genome sequencing in chinese patients. Front Med. (2023) 17:889–906. doi: 10.1007/s11684-023-0994-x 37418076

[53] Lo YT Lim VY Ng M Tan YH Chiang J Chang EWY. A prognostic model using post-steroid neutrophil-lymphocyte ratio predicts overall survival in primary central nervous system lymphoma. Cancers. (2022) 14(7):1818. doi: 10.3390/cancers14071818 35406590 PMC8997514

[54] Guo D Hong L Ji H Jiang Y Lu L Wang X. The mutation of btg2 gene predicts a poor outcome in primary testicular diffuse large B-cell lymphoma. J Inflammation Res. (2022) 15:1757–69. doi: 10.2147/jir.S341355 PMC8923029 35300216

[55] Liu YZ Luo P Liu C Xue K Jin J Xia ZG. Prognostic significance of ldh ratio in serum/cerebral spinal fluid of patients with primary testicular diffuse large B-cell lymphoma. OncoTargets Ther. (2019) 12:10469–75. doi: 10.2147/ott.S228746 PMC6897509 31819527

[56] Shen Y Wang L Ou J Wang B Cen X. Loss of 5-hydroxymethylcytosine as a poor prognostic factor for primary testicular diffuse large B-cell lymphoma. Int J Med Sci. (2022) 19:225–32. doi: 10.7150/ijms.65517 PMC8795795 35165508

[57] Hatzl S Posch F Schulz E Gornicec M Deutsch A Beham-Schmid C. The role of immunohistochemical overexpression of P53 as adverse prognostic factor in primary testicular diffuse large B cell lymphoma. Pathol Oncol Res: POR. (2020) 26:2831–33. doi: 10.1007/s12253-020-00864-6 32602002

[58] Zhang W Yang P Yang Y Liu S Xu Y Wu C. Genomic landscape and distinct molecular subtypes of primary testicular lymphoma. J Trans Med. (2024) 22:414. doi: 10.1186/s12967-024-05140-8 PMC11064289 38693538

[59] Leivonen SK Pollari M Brück O Pellinen T Autio M Karjalainen-Lindsberg ML. T-cell inflamed tumor microenvironment predicts favorable prognosis in primary testicular lymphoma. Haematologica. (2019) 104:338–46. doi: 10.3324/haematol.2018.200105 PMC6355505 30237271

[60] Tsubota K Usui Y Goto H. Identification of prognostic markers in patients with primary vitreoretinal lymphoma by clustering analysis using clinical data. J Clin Med. (2020) 9(7):2298. doi: 10.3390/jcm9072298 32698394 PMC7409000

[61] Zhang Y Li Y Zhuang Z Wang W Wei C Zhao D. Preliminary evaluation of zanubrutinib-containing regimens in dlbcl and the cerebrospinal fluid distribution of zanubrutinib: A 13-case series. Front Oncol. (2021) 11:760405. doi: 10.3389/fonc.2021.760405 35004280 PMC8739956

[62] Yu H Kong H Li C Dong X Wu Y Zhuang Y. Bruton’s tyrosine kinase inhibitors in primary central nervous system lymphoma-evaluation of anti-tumor efficacy and brain distribution. Trans Cancer Res. (2021) 10:1975–83. doi: 10.21037/tcr-21-50 PMC8798964 35116520

[63] Van Bussel MTJ Beijnen JH Brandsma D. Intracranial antitumor responses of nivolumab and ipilimumab: A pharmacodynamic and pharmacokinetic perspective, a scoping systematic review. BMC Cancer. (2019) 19:519. doi: 10.1186/s12885-019-5741-y 31146733 PMC6543612

[64] Ogiya D Murayama N Kamiya Y Saito R Shiraiwa S Suzuki R. Low cerebrospinal fluid-to-plasma ratios of orally administered lenalidomide mediated by its low cell membrane permeability in patients with hematologic Malignancies. Ann Hematol. (2022) 101:2013–19. doi: 10.1007/s00277-022-04893-w 35732975

[65] Li Z Qiu Y Personett D Huang P Edenfield B Katz J. Pomalidomide shows significant therapeutic activity against cns lymphoma with a major impact on the tumor microenvironment in murine models. PloS One. (2013) 8:e71754. doi: 10.1371/journal.pone.0071754 23940785 PMC3734315

[66] Bai S-J He J-X Zheng Y-J Geng Y Gao Y-N Zhang C-X. Clinical characteristics and prognosis of patients with newly diagnosed primary central nervous system lymphoma: A multicenter retrospective analysis. Ann Hematol. (2024) 103(11):4649–60. doi: 10.1007/s00277-024-05797-7 38761184

[67] Yang C Cui Y Ren X Li M Yu K Shen S. Orelabrutinib combined with lenalidomide and immunochemotherapy for relapsed/refractory primary central nervous system lymphoma: A retrospective analysis of case series. Front Oncol. (2022) 12:901797. doi: 10.3389/fonc.2022.901797 35785180 PMC9243261

[68] Ma J Lin Z Ding T Li Q Zhang M Kang H. Pemetrexed plus lenalidomide for relapsed/refractory primary central nervous system lymphoma: A prospective single-arm phase ii study. Front Oncol. (2022) 12:938421. doi: 10.3389/fonc.2022.938421 35898888 PMC9309305

[69] Yonezawa H Narita Y Nagane M Mishima K Terui Y Arakawa Y. Three-year follow-up analysis of phase 1/2 study on tirabrutinib in patients with relapsed or refractory primary central nervous system lymphoma. Neuro-oncol Adv. (2024) 6:vdae037. doi: 10.1093/noajnl/vdae037 PMC11059299 38690230

[70] Fox CP Ali AS Mcilroy G Thust S Martinez-Calle N Jackson AE. A phase 1/2 study of thiotepa-based immunochemotherapy in relapsed/refractory primary cns lymphoma: the tier trial. Blood Adv. (2021) 5:4073–82. doi: 10.1182/bloodadvances.2021004779 PMC8945638 34464973

[71] Tun HW Johnston PB Deangelis LM Atherton PJ Pederson LD Koenig PA. Phase 1 study of pomalidomide and dexamethasone for relapsed/refractory primary cns or vitreoretinal lymphoma. Blood. (2018) 132:2240–48. doi: 10.1182/blood-2018-02-835496 PMC6265643 30262659

[72] Ghesquieres H Chevrier M Laadhari M Chinot O Choquet S Moluçon-Chabrot C. Lenalidomide in combination with intravenous rituximab (Revri) in relapsed/refractory primary cns lymphoma or primary intraocular lymphoma: A multicenter prospective ‘Proof of concept’ Phase ii study of the french oculo-cerebral lymphoma (Loc) network and the lymphoma study association (Lysa)†. Ann Oncol. (2019) 30:621–28. doi: 10.1093/annonc/mdz032 30698644

[73] Yu W Huang L Mei H Li Y Niu T Zou D. Real-world experience of commercial relmacabtagene autoleucel (Relma-cel) for relapsed/refractory central nervous system lymphoma: A multicenter retrospective analysis of patients in China. J Immunother Cancer. (2024) 12(5):e008553. doi: 10.1136/jitc-2023-008553 38802271 PMC11131121

[74] Mercadal S Ahn KW Allbee-Johnson M Ganguly S Geethakumari PR Hong S. Outcomes of patients with primary central nervous system lymphoma following cd19-targeted chimeric antigen receptor T-cell therapy. Haematologica. (2024) 110(1):218–21. doi: 10.3324/haematol.2024.285613 PMC11694114 39234871

[75] Frigault MJ Dietrich J Gallagher K Roschewski M Jordan JT Forst D. Safety and efficacy of tisagenlecleucel in primary cns lymphoma: A phase 1/2 clinical trial. Blood. (2022) 139:2306–15. doi: 10.1182/blood.2021014738 PMC9012129 35167655

[76] Karschnia P Arrillaga-Romany IC Eichler A Forst DA Gerstner E Jordan JT. Neurotoxicity and management of primary and secondary central nervous system lymphoma after adoptive immunotherapy with cd19-directed chimeric antigen receptor T-cells. Neuro-oncology. (2023) 25:2239–49. doi: 10.1093/neuonc/noad118 PMC10708936 37402650

[77] Lacan C Caron J Tarantino N Fouquet B Cherai M Parizot C. Car T-cell therapy for central nervous system lymphomas: blood and cerebrospinal fluid biology, and outcomes. Haematologica. (2023) 108:3485–90. doi: 10.3324/haematol.2023.282875 PMC10690903 37345469

