# Supplementary material for: Advances in primary large B-cell lymphoma of immune-privileged sites
Source: Front Immunol. 2025 Feb 26;16:1533444. doi: 10.3389/fimmu.2025.1533444 (PMC11896999; doi:10.3389/fimmu.2025.1533444)
Supplement: Supplementary Table 1 — Pathological, molecular, and genetic changes of IP-LBCL. *DLBCL accounted for 95% of PCNSL, and the remaining pathological types accounted for 5%. **DLBCL accounted for 80-90% of PCNSL, and the remaining pathological types accounted for 10-20%. [file Table1.docx]

| Supplementary Table: Pathological, molecular, and genetic changes of IP-LBCL. | | | | | | |
| --- | --- | --- | --- | --- | --- | --- |
|  | **CNS** | **Testicles** | **Vitreoretinal** | **Intravascular** | **Skin** | **Breast or**  **adrenal gland** |
| Pathological type |  |  |  |  |  |  |
|  | DLBCL* | DLBCL** | DLBCL | DLBCL | DLBCL | DLBCL |
|  | MZL | BL |  |  |  |  |
|  | ALCL | MCL |  |  |  |  |
|  | BL | T/NK |  |  |  |  |
|  | LBL | FL |  |  |  |  |
|  | T/NK |  |  |  |  |  |
|  | HL |  |  |  |  |  |
| Genetic changes |  |  |  |  |  |  |
| Common |  | | | | | |
|  | MYD88 and CD79B | | | | | |
| Signaling pathway |  |  |  |  |  |  |
|  | CDKN2A | CDKN2A | CDKN2 | PIM1 | CDKN2A | CDKN2A/B |
|  | PIM1 | NFKBIZ | PIM1 | ETV6 | NFKBIE | Etc. |
|  | IFR4 | BCL-2 | IGLL5 | IRF4 | REL |  |
|  | CARD11 | BCL-6 | TBL1XR1 | TMEM30A | BRAF |  |
|  | PRDM1 | LILRA3 | ETV6 | BTG2 | MED12 |  |
|  | DDX3X | SP1B | Etc. | NOTCH2 | PIK3R1 |  |
|  | ATG5 | BCL2L12 |  | CCND3 | STAT3 |  |
|  | TNFPAI13 | PAK4 |  | GNA13 | Etc. |  |
|  | MALT1 | PPP5C |  | IRF4 |  |  |
|  | BCL-6 | FIZ1 |  | Etc. |  |  |
|  | ATM | FOXP1 |  |  |  |  |
|  | TP53 | MALT1 |  |  |  |  |
|  | PTEN | BCL10 |  |  |  |  |
|  | PIK3CA | MDM2 |  |  |  |  |
|  | JAK3 | YEATS4 |  |  |  |  |
|  | CTNNB1 | MALT1 |  |  |  |  |
|  | PTPN1 | Etc. |  |  |  |  |
|  | KRAS |  |  |  |  |  |
|  | Etc. |  |  |  |  |  |
| Immune escape |  |  |  |  |  |  |
|  | HLA-D | HLA | - | HLA-B | HLA | PD-L1 |
|  | PD-L1/2 | PD-L1/2 |  | PD-L1/2 | CIITA | Etc. |
|  | Etc. | CIITA |  | Etc. | B2M |  |
|  |  | B2M |  |  | Etc. |  |
|  |  | Etc. |  |  |  |  |

*DLBCL accounted for 95% of PCNSL, and the remaining pathological types accounted for 5%.

**DLBCL accounted for 80-90% of PCNSL, and the remaining pathological types accounted for 10-20%.
